# Insecticide Resistance in Areas under Investigation by the International Centers of Excellence for Malaria Research: A Challenge for Malaria Control and Elimination

**DOI:** 10.4269/ajtmh.14-0844

**Published:** 2015-09-02

**Authors:** Martha L. Quiñones, Douglas E. Norris, Jan E. Conn, Marta Moreno, Thomas R. Burkot, Hugo Bugoro, John B. Keven, Robert Cooper, Guiyun Yan, Angel Rosas, Miriam Palomino, Martin J. Donnelly, Henry D. Mawejje, Alex Eapen, Jacqui Montgomery, Mamadou B. Coulibaly, John C. Beier, Ashwani Kumar

**Affiliations:** Public Health Department, Faculty of Medicine, National University of Colombia, Bogota, Colombia; Department of Molecular Microbiology and Immunology, Johns Hopkins Malaria Research Institute, Johns Hopkins University Bloomberg School of Public Health, Baltimore, Maryland; Wadsworth Center, New York State Department of Health, Albany, New York; Department of Biomedical Sciences, School of Public Health, State University of New York, Albany New York; Division of Infectious Diseases, School of Medicine, University of California, San Diego, California; James Cook University, Queensland Tropical Health Alliance, Queensland, Australia; National Vector Borne Disease Control Programme, Ministry of Health, Honiara, Solomon Islands; Vector Borne Disease Unit, Papua New Guinea Institute of Medical Research, Madang, Papua New Guinea; Australian Army Malaria Institute, Gallipoli Barracks, Queensland, Australia; Program in Public Health, University of California, Irvine, California; Instituto de Medicina Tropical, Alexander von Humboldt, Universidad Peruana Cayetano Heredia, Lima, Peru; Instituto Nacional de Salud, Lima, Peru; Department of Vector Biology, Liverpool School of Tropical Medicine, Liverpool, United Kingdom; Infectious Diseases Research Collaboration, Kampala, Uganda; National Institute of Malaria Research, Chennai, India; Department of Entomology, Pennsylvania State University, University Park, Pennsylvania; Malaria Research and Training Centre, Faculty of Medicine and Dentistry, University of Mali, Bamako, Mali; Department of Public Health Sciences, University of Miami Miller School of Medicine, Miami, Florida; National Institute of Malaria Research, Goa, India

## Abstract

Scale-up of the main vector control interventions, residual insecticides sprayed on walls or structures and/or impregnated in bed nets, together with prompt diagnosis and effective treatment, have led to a global reduction in malaria transmission. However, resistance in vectors to almost all classes of insecticides, particularly to the synthetic pyrethroids, is posing a challenge to the recent trend of declining malaria. Ten International Centers of Excellence for Malaria Research (ICEMR) located in the most malaria-endemic regions of the world are currently addressing insecticide resistance in the main vector populations, which not only threaten hope for elimination in malaria-endemic countries but also may lead to reversal where notable reductions in malaria have been documented. This communication illustrates the current status of insecticide resistance with a focus on the countries where activities are ongoing for 9 out of the 10 ICEMRs. Most of the primary malaria vectors in the ICEMR countries exhibit insecticide resistance, albeit of varying magnitude, and spanning all mechanisms of resistance. New alternatives to the insecticides currently available are still to be fully developed for deployment. Integrated vector management principles need to be better understood and encouraged, and viable insecticide resistance management strategies need to be developed and implemented.

## Introduction

The fight against malaria between 2000 and 2012 has resulted in global reductions of 42% in mortality and 25% in incidence of malaria.^1^ This achievement can be attributed to the introduction of artemisinin-based combination therapies and improvement in diagnosis, but also to the major scale-up of vector control interventions, such as the mass distribution of long-lasting insecticide-treated nets (LLINs) and indoor residual spraying (IRS). The World Health Organization (WHO) recommends that in areas where malaria transmission is targeted by vector control, every person at risk should be protected by either LLINs or IRS. This goal is still to be achieved, but in the last decade, the global distribution of LLINs has increased considerably. For example, in the sub-Saharan African region, from 2010 to 2013, an estimated 443 million LLINs have been delivered, reaching a coverage of up to 60%, a dramatic improvement from only 10% coverage in 2000.^1^

A major threat for malaria control programs worldwide is the development and spread of insecticide resistance in vector populations. Unfortunately, the use of insecticides for both public health and agriculture has induced selective pressure(s) on numerous insect populations, including *Anopheles* mosquitoes involved in malaria parasite transmission, resulting in the selection of highly resistant vector populations. According to the WHO, insecticide resistance is defined as the ability of an insect to withstand the effects of an insecticide by becoming resistant to its toxic effects by means of natural selection and mutations.^2^ Many malaria vector species have acquired multiple insecticide resistance as they have been exposed to a battery of insecticides since the eradication era of the 1950s. The reliance on insecticides to reduce vectorial capacity for malaria transmission control is thus facing a grave threat and becoming a major public health concern. The common mechanisms by which vectors acquire insecticide resistance are metabolic resistance (e.g., glutathione S-transferases, esterases, monooxygenases), target site resistance (e.g., mutations in acetylcholinesterase gamma-aminobutyric acid receptors, insensitivity of the sodium channels—*kdr*, or knock down resistance), reduced penetration, and behavioral avoidance.^3^

The goal of this communication is to review the current situation regarding insecticide resistance in the regions under study by nine out of the 10 National Institutes of Health (NIH) International Centers for Excellence for Malaria Research (ICEMRs). The ICEMRs have activity on all continents with malaria transmission; Africa has four ICEMRs: west Africa (Mali, Senegal, and The Gambia), Uganda (Uganda), southern Africa (Zambia and Zimbabwe), and Mali (Mali), from which the former three contributed to this manuscript. Latin America (LA) has two ICEMRs: LA (Guatemala, Panama, Colombia, and Perú) and Amazonia (Peru and Brazil). Asia has three ICEMRs: India (India), south Asia (India), and southeast (SE) Asia (China, Thailand, and Myanmar). These investigations of malaria transmission and control in the ICEMR network provide an ideal opportunity to broadly examine global trends in insecticide resistance in the context of viable strategies for malaria elimination.

## Bioassay Methods for Detecting Insecticide Resistance

The methods for evaluation of the status of susceptibility in *Anopheles* mosquitoes have been proposed and standardized by the WHO, using papers impregnated with the diagnostic dosage and exposure time for each insecticide,^4^ and/or using the United States Centers for Disease Control (CDC) methods of coated bottles.^5^ The standardized WHO protocol^4^ was used by all ICEMRs. Briefly, each bioassay consists of insecticide-exposed tubes, usually four, and a control tube with no insecticide. In each tube, 20–25 adult female mosquitoes are exposed for 1 hour to any insecticide to be evaluated, except the organophosphate (OP) fenitrothion, for which the exposure time is 2 hours. Mortality is recorded 24 hours postexposure. Some ICEMRs, such as the LA and Amazonian ICEMRs, used both WHO and CDC bottle bioassay because national malaria control programs use the bottle bioassay as a routine method, due to difficulties in the acquisition of the WHO kits. Bottles are prepared following the CDC (WHO approved) protocol. Each population is evaluated with diagnostic doses and times previously established for *Anopheles* species.^5^ About 15–20 non-blood-fed females from each site and species are exposed to the diagnostic dose for each insecticide to be evaluated, in coated 250 mL glass bottles. Each test consists of four treated bottles and one control bottle coated with acetone or ethanol only. Mortality is recorded every 15 minutes for a 2-hour period. Mortality criteria include mosquitoes with difficulties in flying or standing on the bottle surface. A susceptible population as determined by either method will be killed when exposed to the insecticides for the diagnostic period. Mortalities lower than 98% suggest the existence of resistance in the population^4^ and often serve as an early warning.

A review of tests carried out in each of the ICEMR countries was compiled. Each ICEMR provided its own data, with a few records previously published. The data have been compiled and are presented by geographical region, with the proportion of mosquito mortality/susceptibility illustrated by country and vector species.

## Current Status of Insecticide Resistance in ICEMR Regions

### African region.

The vast majority (80%) of malaria cases and deaths (90%) from the entire world occur in the African region every year.^1^ In the last decade, vector control has been intensified, by the use of insecticides in LLINs or IRS, which has led to increased insecticide pressure on the vector populations. *Anopheles gambiae* s.s. and *Anopheles funestus* s.s., two of the most important malaria vectors in sub-Saharan Africa and therefore globally, have been subjected to closely monitored evaluations for changes in susceptibility to all insecticides of public health use, given the devastating potential consequences of insecticide resistance in these species. Resistance to pyrethroids (PY), the main insecticide group currently used for malaria control, is now widespread in African vectors.^6^

**Figure 1.**
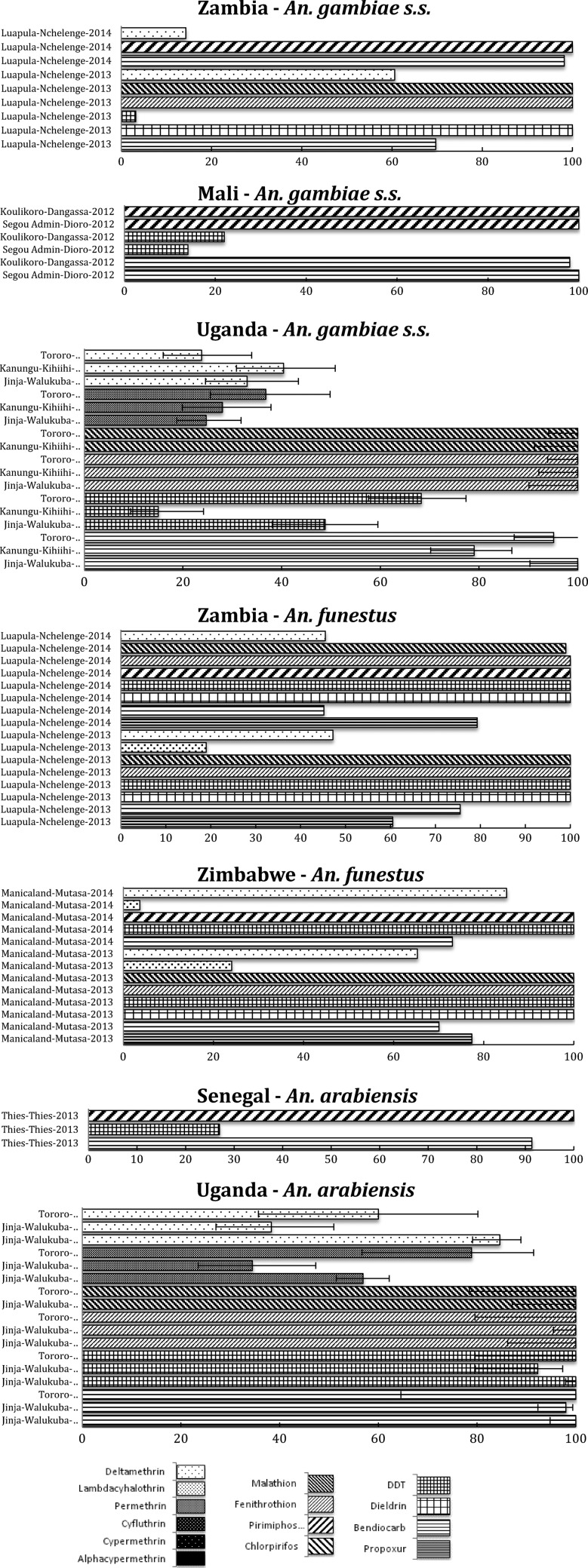
African region, including countries in the southern Africa, east Africa, and west Africa International Centers of Excellence for Malaria Research: summary of insecticide susceptibility status of malaria vectors showing the proportion of mosquitos killed in susceptibility bioassay tests, by country and site.

At the ICEMR study sites in Zambia, *An. gambiae* s.s. is still completely susceptible to OP (malathion and fenitrothion) and the organochlorines (OC) (dichlorodiphenyltrichloroethane [DDT] and dieldrin) (Figure 1

). However, strong resistance to DDT has been illustrated elsewhere in the country, and high levels of PY are widespread.^7^

*Anopheles funestus* s.s. has shown similar resistance profiles in Zambia and Zimbabwe, being completely susceptible to DDT, dieldrin, and OPs (malathion, fenitrothion, and pirimiphos-methyl), but showing resistance to PYs (deltamethrin and lambda-cyhalothrin) and the carbamates (C) (bendiocarb and propoxur)^8^,^9^ (Figure 1).

The third major African malaria vector, *Anopheles arabiensis*, currently present at only one of the ICEMR sites in southern Zambia, has been reported as fully susceptible to all classes of insecticides at this site.^8^

In west Africa, the two major malaria vectors *An. gambiae* s.s. and *An. funestus* s.s. have previously been found to be potentially resistant to PY and DDT.^10^–^12^ In Senegal, an increase in the frequency of the *kdr* mutation in *An. gambiae* s.s. was observed when examined before and after the introduction of LLINs.^13^ Since then, that population of *An. gambiae* s.s. has shown resistance to DDT and PY insecticides (deltamethrin, lambda-cyhalothrin, and permethrin), with mortality rates ranging from 46% to 63%, but it remains completely susceptible to fenitrothion (OP) and bendiocarb (C).^13^ The use of pyrethroids as pesticides in agriculture and for bed net treatment has been recognized as a factor responsible for the selection of resistant mosquitoes in sub-Saharan Africa.^14^,^15^

Tests carried out in Mali (Segou and Koulikoro regions) and Senegal (Thies region) showed high resistance levels to DDT in both *An. gambiae* s.s. and *An. arabiensis*, but susceptibility to bendiocarb (C) and pirimiphos-methyl (OP) (Figure 1). Any carbamate or OP could be introduced for IRS to replace pyrethroids for IRS as part of an insecticide resistance management (IRM) strategy in this region.

In the Uganda ICEMR, *An. gambiae* s.s. and *An. arabiensis* have been found to occur in sympatry at all the three sites, namely Jinja, Tororo, and Kanungu, with the highest levels of *An. arabiensis* species composition (approximately 80% of all mosquitoes collected) found in Jinja.^16^ High levels of DDT, deltamethrin, and permethrin resistance have been observed in *An. gambiae* s.s. in Jinja,^16^ Tororo,^17^ and Kanungu (Figure 1). In contrast, no resistance to DDT has been observed in *An. arabiensis* from Jinja or Tororo (there is a very low abundance of *An. arabiensis* in Kanungu). However, resistance to deltamethrin and permethrin has been observed in *An. arabiensis* from both Jinga and Tororo. There is new evidence of incipient bendiocarb (C) resistance in two of the Uganda ICEMR sites, namely Kanungu and Tororo. This is of particular concern and a major challenge to the IRS campaign and further exacerbates the challenge of pyrethroid resistance at these sites.

### LA region.

**Figure 2.**
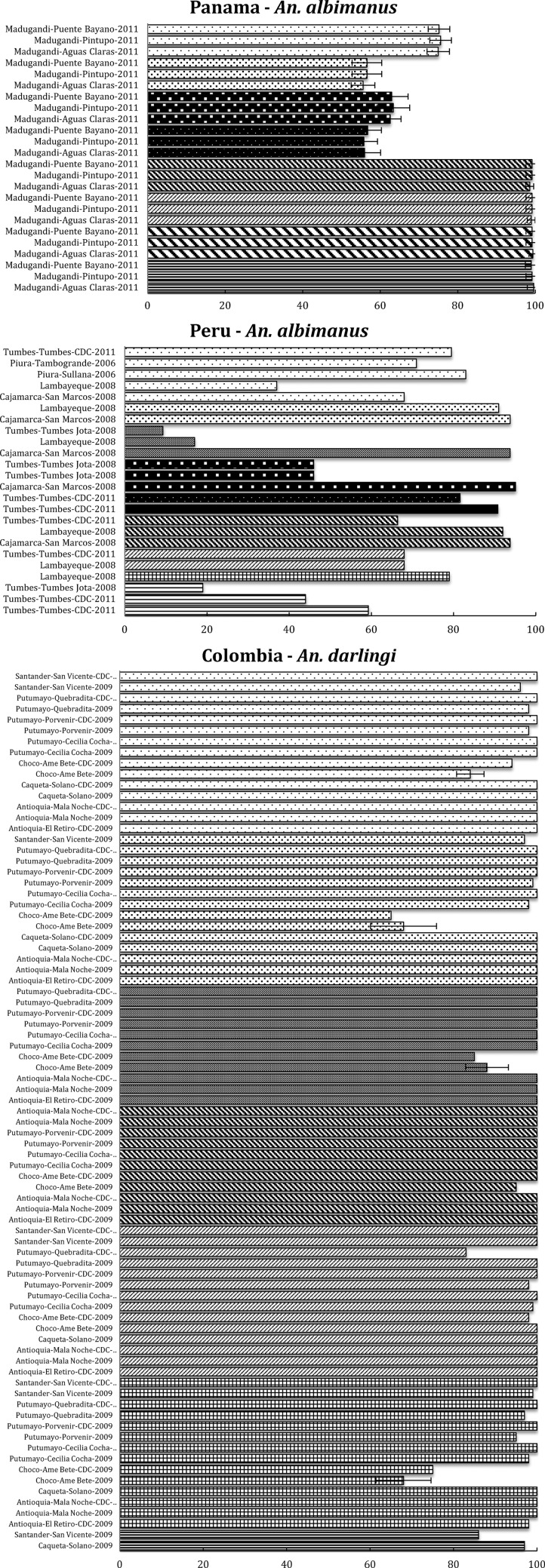

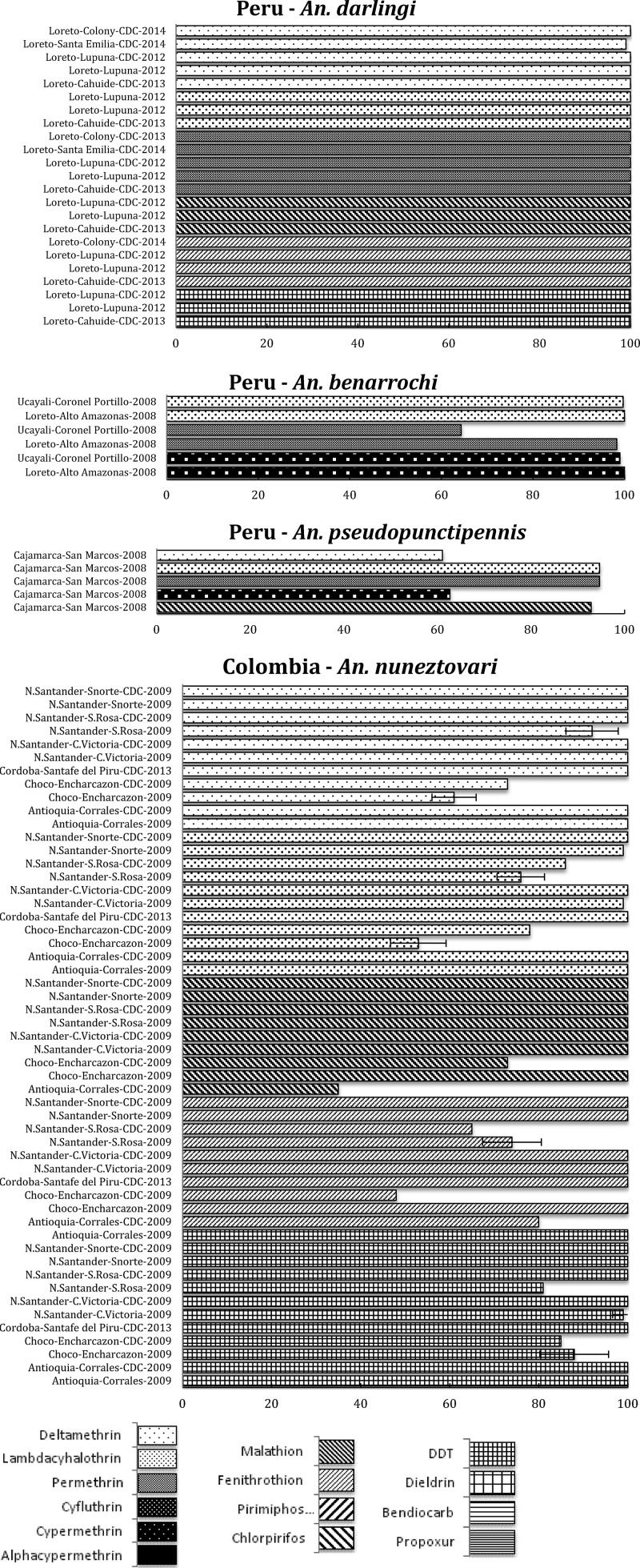
Latin American (LA) region, including countries in the LA and Amazonia International Centers of Excellence for Malaria Research: summary of insecticide susceptibility status of malaria vectors showing the proportion of mosquitos killed in susceptibility bioassay tests, by country and site.

*Anopheles darlingi*, the main malaria vector in LA, and particularly responsible for malaria transmission in the Amazon region, is generally susceptible to all insecticides throughout its distribution. However, a population in western Colombia (Choco) exhibited DDT resistance in the 1990s,^18^ and to DDT and PY when resampled in 2005–2009 (permethrin, lambda-cyhalothrin, and deltamethrin).^19^ Despite this resistance to DDT and PY, this population showed susceptibility to OP (malathion and fenitrothion). Apart from this particular population, in the Amazon region of Perú-Brazil (Amazonian ICEMR), along the Pacific coast (LA ICEMR) and in other areas in LA,^19^,^20^ this species shows complete insecticide susceptibility (Figure 2

).

*Anopheles albimanus* has also been subjected to insecticide resistance surveillance throughout its range in the Americas. In Central America, resistance to a variety of insecticides was reported in 1970 and associated mainly with insecticide use in agriculture.^21^ In Panama, this species has demonstrated resistance to PYs (cyfluthrin, cypermethrin, deltamethrin, and lambda-cyhalothrin), and susceptibility to OPs (malathion and fenitrothion)^22^ (Figure 2). In contrast, this species has shown an alarming resistance to all insecticides evaluated in northwestern coastal Peru. As shown in Figure 2, lower than 95% mortality rates have been recorded for the bendiocarb (C), OCs such as DDT, OPs (malathion and fenitrothion), and all PYs evaluated (permethrin, cyfluthrin, cypermethrin, deltamethrin, and lambda-cyhalothrin).^23^ The distribution pattern of *An. albimanus* in Peru overlaps with rice cultivation areas where insecticides are used frequently, and it is likely that this pressure has influenced the selection of resistance in vector populations.

*Anopheles nuneztovari* s.l. is one of the main vectors of malaria in Colombia and Venezuela. This species has been described as endophagic but exophilic, with a tendency to avoid contact with walls sprayed with insecticides. In a series of evaluations conducted in Colombia, this species exhibited insecticide resistance in a population on the border between Colombia and Venezuela to PYs, OPs, and DDT^24^ (Figure 2). Similarly, *Anopheles benarrochi* and *Anopheles pseudopunctipennis* are considered of importance as vectors in Peru.^25^ *Anopheles benarrochi* is susceptible to PY, except for a population from Ucayali (on the border with Brazil) that demonstrated resistance to permethrin, whereas *An. pseudopunctipennis* from Cajamarca, in the northwest of the country has shown less than 95% mortality for all insecticides tested (permethrin, deltamethrin, lambda-cyhalothrin, cypermethrin, and malathion) (Figure 2).

In general, resistance in vectors in LA is focal, probably due to insecticide pressure from agriculture use. Most importantly, at this time, the primary vector in LA, *An. darlingi*, has shown susceptibility through most of its distribution, except for one population in Colombia.

Given the local availability of supplies for the CDC bioassay, this methodology is performed in Colombia every year at sentinel sites selected by the insecticide resistance surveillance network (IRSN).^26^ In Peru, this surveillance is based on the WHO bioassay, but in some localities the CDC bottle bioassay has also been used by the malaria control program^27^ and Amazonian ICEMR. CDC results compatible with suspected resistance are confirmed by WHO methodology whenever possible.^28^ Although both methods report percentage mortalities, the results from the CDC bottle bioassay are not directly comparable with those obtained from the WHO susceptibility tube test even though both methods have been shown to reliably identify insecticide resistance where it occurs.^4^

To directly compare these methods, WHO and CDC tests were conducted simultaneously on the same mosquito populations from 64 localities in Colombia^19^,^24^ and Peru. Following the new WHO criteria of mortalities, below 98% being suggestive of the existence of resistance,^4^ these two tests gave identical results in 84.4% (54/64) of the comparisons, identifying 45 susceptible and 9 resistant populations. The remaining 15.6% (10/64) of comparisons did not match; in six populations, the mortality rate by the WHO method was between 81% and 97%, whereas for the CDC bottle bioassay it was 100%; and in four populations, 100% mortality was obtained using the WHO test, but the CDC bottle bioassay mortalities ranged between 48% and 83%. The Kappa index for the 64 locality comparisons was 0.544, interpreted as moderate agreement between the methods.^29^ Whenever there are discrepancies between methods, IRSN recommends synergists be used together with biochemical methods for confirmation and determination of the possible resistance mechanisms in that particular population.^28^ Despite the discrepancies noted above, either method can be used in a routine surveillance system for early detection of resistance and to support decisions on the appropriate management of vector populations.

### Pacific region.

**Figure 3.**
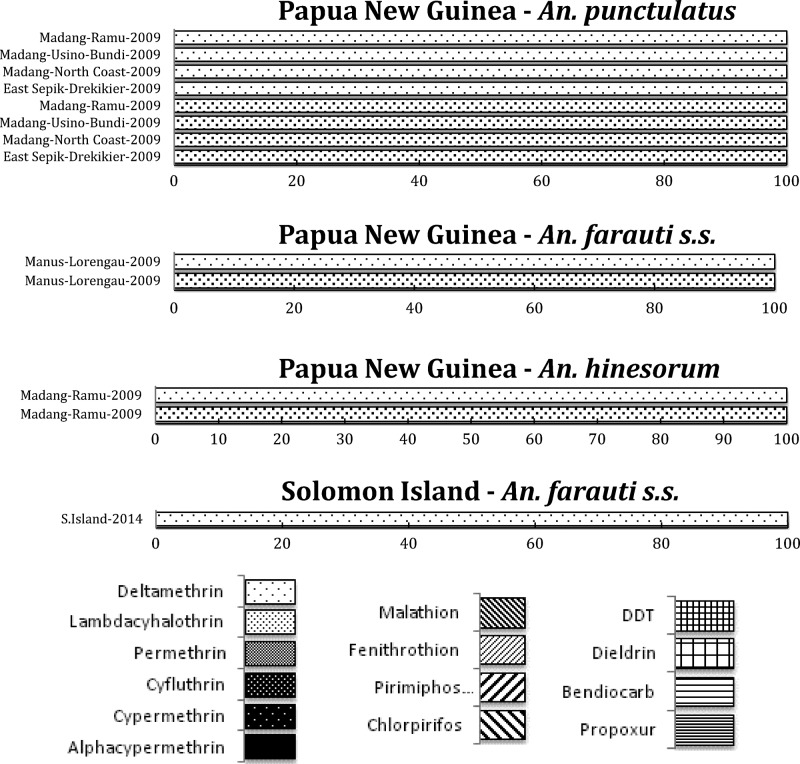
Pacific region, including countries in the Pacific International Centers of Excellence for Malaria Research: summary of insecticide susceptibility status of malaria vectors showing the proportion of mosquitos killed in susceptibility bioassay tests, by country and site.

*Anopheles farauti* s.l. populations composed of *An. farauti*, *Anopheles punctulatus*, and *Anopheles hinesorum* from five study sites in the Madang, Manus, and east Sepik provinces in Papua New Guinea were tested for susceptibility to the PYs deltamethrin and lambda-cyhalothrin and the presence of the *kdr* allele.^30^ All populations (*An. farauti* s.s. in Manus and two sites in Madang, *An. punctulatus* in east Sepik and anophelines composed of both *An. hinesorum* and *An. punctulatus*) were 100% susceptible (Figure 3

) with no detection of the *kdr* allele. Further screening for the *kdr* genotype in wild-caught adult *An. punctulatus* s.l. (*N* = 90) collected from seven different PNG provinces did not detect *kdr* allele in any of the *An. punctulatus* species.^31^

Similar results with the WHO paper test were found in 2014 in the Solomon Islands where 100% susceptibility to deltamethrin was shown in *An. farauti* s.s. from the western Province (Cooper, unpublished), Temotu, Central, Choiseul, and Malaita (Bugoro, unpublished). However, in 2013, moderate resistance to lambda-cyhalothrin was found in Malaita and Central provinces, to permethrin in Central and Guadalcanal provinces, and to deltamethrin in Guadalcanal (Bugoro, unpublished).

The absence of high levels of resistance recorded in many geographic areas is not surprising given the well-documented development of behavioral resistance in *An. farauti* s.l. following exposure to DDT used in IRS in both Papua New Guinea and the Solomon Islands.^32^,^33^ The behavioral resistance phenotype observed is a shift toward earlier feeding with a higher proportion of feeds occurring outdoors. Such feeding shifts prevent insecticide exposure of the vector to IRS-treated walls and/or pyrethroids in insecticide-treated nets.^34^

### South Asia (India).

India has six primary vectors of malaria; *Anopheles culicifacies*, *Anopheles stephensi*, *Anopheles fluviatilis*, *Anopheles minimus*, *Anopheles dirus* (*Anopheles baimai*), and *Anopheles sundaicus*. The first three species have been subjects for determination of insecticide susceptibility and are responsible for most of the malaria transmission in the region. There are two ICEMRs in India. The first is Malaria Evolution in South Asia with operational sites in Goa, Wardha (Maharashtra), Ranchi (Jharkhand), and Dibrugarh (Assam) where *An. stephensi*, *An. culicifacies*, *An. minimus*, and *An. dirus* (*An. baimai*) are the main vectors. The second ICEMR is Center for the Studies of Complex Malaria in India with an urban site, Chennai, with the urban malaria vector *An. stephensi*, and two rural sites in Gujarat and Odisha states where *An. culicifacies* and *An. fluviatilis* are the main vectors.

**Figure 4.**
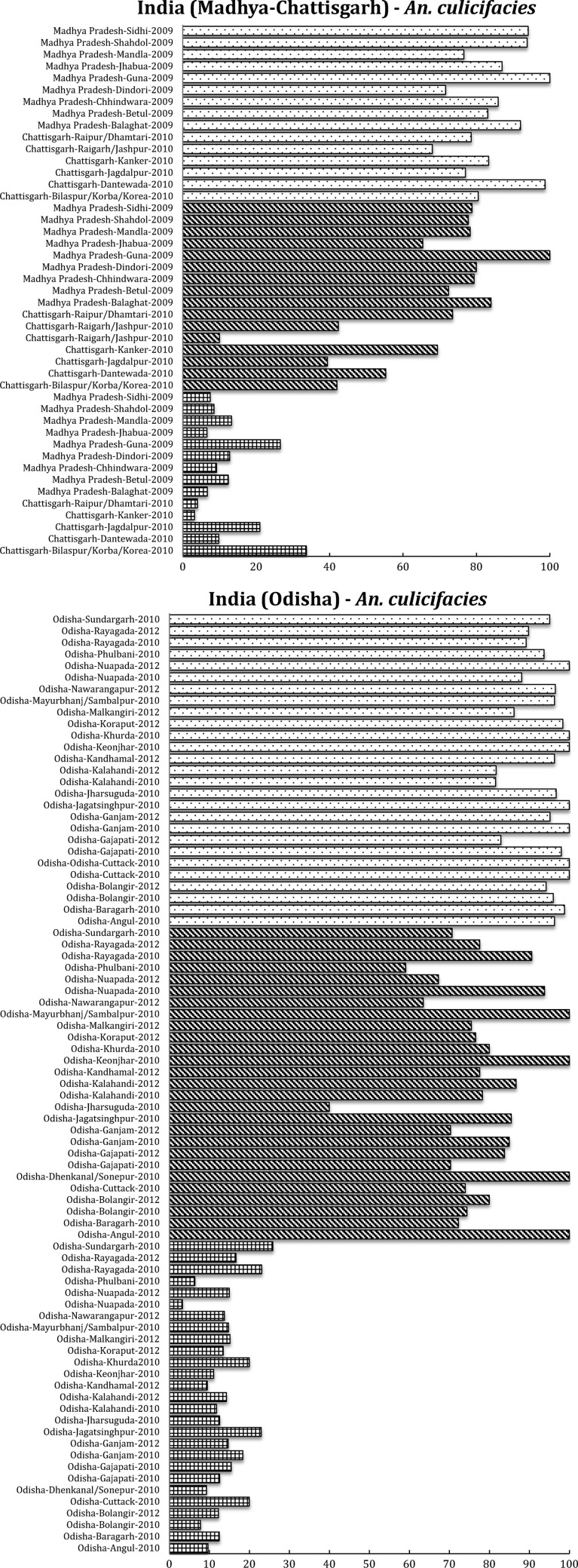

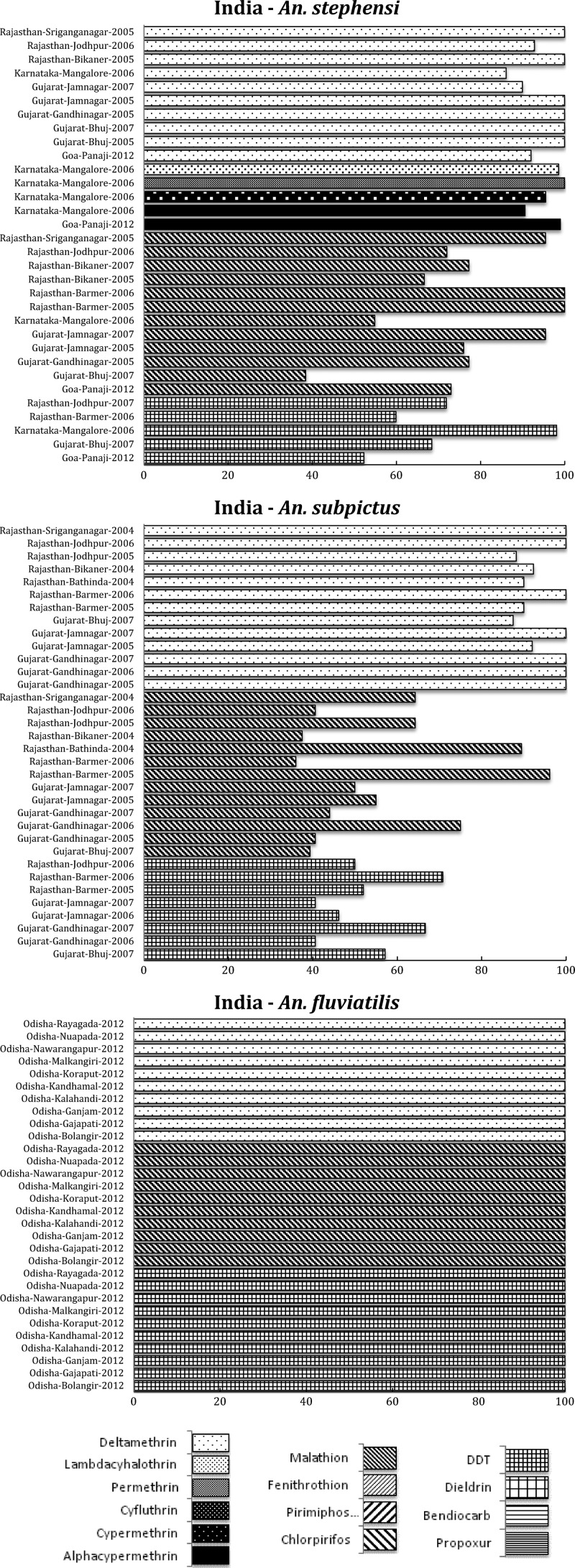
South Asian region, including India and south Asia International Centers of Excellence for Malaria Research: summary of insecticide susceptibility status of malaria vectors showing the proportion of mosquitos killed in susceptibility bioassay tests, by country and site.

*Anopheles culicifacies* is resistant to DDT and malathion in most districts of Odisha and in other states, and highly resistant to deltamethrin although a few regions retain sensitive populations.^35^,^36^ In general, the problem of DDT resistance in *An. culicifacies* is acute in Odisha, Madhya Pradesh, and Chhattisgarh (Figure 4

). In Odisha, a highly malarious state of India, the resistance to deltamethrin in *An. culicifacies* is increasing, whereas other populations remain fully susceptible.^36^ In Chhattisgarh state, resistance to deltamethrin in *An. culicifacies* is also increasing. In Tamil Nadu, the susceptibility status of *An. culicifacies* from Rameswaram Island to deltamethrin (0.05%) and cyfluthrin (0.15%) was compared with the strains from Subbareddipalayam, an adjoining area located in the northern outskirts of Chennai city. Another study in Rameswaram Island in Tamil Nadu, has also reported reduced susceptibility to deltamethrin in *An. culicifacies*.^37^ In a study in Surat, Gujarat, development of pyrethroid resistance has been reported in sibling species, B and C of *An. culicifacies*.^38^ In contrast, the sibling species *An. fluviatilis* S is also a dominant vector in Odisha. So far, *An. fluviatilis* remains completely susceptible to all insecticides including pyrethroids and DDT used for vector control and OPs used for agricultural purposes.^36^

The principal urban vector in India, *An. stephensi*, has been reported to be resistant to malathion in Goa,^39^ whereas low-level resistance as well as complete susceptibility have been observed in two native populations in Rajasthan. *Anopheles stephensi* was highly susceptible to deltamethrin in Rajasthan and Gujarat, and lambda-cyhalothrin in Karnataka state.^40^ In Goa, resistance to deltamethrin is building, whereas this species was highly resistant to DDT in Rajasthan, Gujarat, and Goa.^41^ In one location in Karnataka state, however, a population of *An. stephensi* was found to be highly susceptible to DDT, illustrating the heterogeneity in these resistance patterns (Figure 4). Bioassays with cyfluthrin showed that > 95% mortality occurred in this species in Karnataka,^42^ whereas in Goa, complete susceptibility was earlier observed to pirimiphos-methyl.^43^

In *An. subpictus*, the vectorial capacity of which is under investigation, widespread DDT (OC) and malathion (OP) resistance was reported from Rajasthan and Gujarat^44^ (Figure 4). Many populations of this species tested in Gujarat and Rajasthan also showed some degree of resistance to deltamethrin (PY). In contrast, in Odisha, complete susceptibility to DDT, malathion, and deltamethrin has been found in *An. fluviatilis*, an important malaria vector in the hills and foothills in India^45^ (Figure 4).

### SE Asia.

In SE Asia, malaria vectors are highly diverse in species composition, population dynamics, ecological niche requirement, host feeding preference, and vector competence.^46^ Malaria vector species in SE Asia exhibit tremendous spatial heterogeneity in distribution. For example, in tropical and sub-tropical regions of China (below 25°N latitude), *An. minimus* s.l. and *An. dirus* s.l. are the main vectors, whereas in more temperate regions (above 33°N latitude), *Anopheles sinensis* is the major malaria vector.^46^ In the areas between 25°N latitude and 33°N latitude, *Anopheles anthropophagus* and *Anopheles liangshanensis* are important regional malaria vectors. In Thailand, in addition to *An. dirus* and *An. minimus*, *Anopheles maculatus* and *Anopheles aconitus* are considered to be primary human malaria vectors.^46^

Reducing vector-human contact by the use of LLINs has been shown to be effective in reducing malaria prevalence in SE Asia.^47^ Along with the use of insecticides to reduce abundance of disease vectors, the application of insecticides for agricultural purposes increases the likelihood and speed at which resistance can develop.^48^

**Figure 5.**
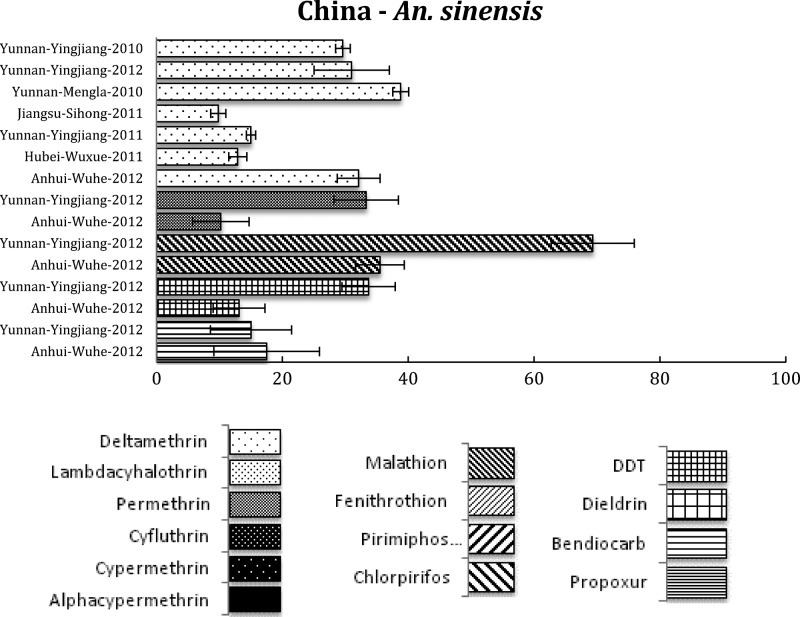
Southeast (SE) Asian region, including countries in the SE Asia International Centers of Excellence for Malaria Research: summary of insecticide susceptibility status of malaria vectors showing the proportion of mosquitos killed in susceptibility bioassay tests, by country and site.

Previous studies in SE Asia suggest a patchy distribution of insecticide resistance in four malaria vector species. Between 1990 and 1997, DDT resistance has been detected in *An. dirus* s.l. and *An. minimus* s.l., and permethrin resistance was also found in a population of *An. minimus* s.l. from northern Thailand.^46^ In Vietnam, pyrethroid-susceptible and pyrethroid-tolerant *An. minimus* populations were found, and *An. minimus* also showed resistance to DDT (OC) and pyrethroids in some sites in Cambodia and Laos.^49^ *Anopheles dirus* s.s., the main vector in forested malaria foci, was permethrin susceptible throughout the Mekong region, but in central Vietnam it showed possible resistance to pyrethroids.^49^ In 2006, resistance to deltamethrin was reported in *An. sinensis* in China.^50^ Recently, extensive and high level of multiple insecticide resistance was found in *An. sinensis* (Figure 5

) from the malaria-endemic areas in China.^51^,^52^ The patchy distribution of resistant genes in the vector population will require resistance monitoring to limit the spread of resistance genes among populations.

## Discussion

With few exceptions, such as the Pacific and Amazon regions, most countries involved with the NIH ICEMR research programs are facing significant problems with insecticide resistance. Of tremendous concern are the growing resistance levels to pyrethroids illustrated in many regions and for the most important malaria vector species. This class of insecticides is currently the only suitable ones for LLINs, and in many regions, pyrethroids are also used for IRS. In most African countries, DDT and PY resistance mediated in part by metabolic resistance and/or *kdr* is widespread,^53^ and occurs even in a relatively short period of time after the introduction of mass distribution campaigns of LLINs, as has been the case in Senegal.^13^ The use of OP or carbamates for IRS seems to be the alternative, which has an economic impact on the malaria control programs not only for the higher cost of these classes of insecticides compared with PY but also for the operational cost due to the need of applications two or three times a year, depending on the transmission seasons.

Some regions, such as the Indian and SE Asian subcontinents, have a diverse range of vector species responsible for malaria transmission of *Plasmodium falciparum*, *Plasmodium vivax*, and *Plasmodium malariae*, with enormously varied resistance patterns. *Anopheles culicifacies* is responsible for approximately 65% of the total malaria cases in India and resistance in this species to almost all classes of insecticide threatens malaria control.^54^ In addition, two of the principal vectors of malaria in India, *An. culicifacies* and *An. stephensi*, are resistant to multiple insecticides although resistance varies dramatically between populations, apparently depending on the history of insecticide use and selection. Some important vectors (e.g., *An. fluviatilis*), however, show remarkable susceptibility to most insecticides, as depicted here (Figure 4), and there is need to investigate the resistance status of other vectors in India, that is, *An. sundaicus*, *An. dirus*, and *An. minimus*. In SE Asia, the situation is more problematic for the main vector *An. sinensis*, since resistance to all classes of insecticides is at high levels and long-term rotational use of various insecticides has led to the selection of this high insecticide resistance.^52^ This is a clear call for urgent development and expansion of non-insecticide-dependent tools for vector management, such as larvivorous fish and source reduction, as used as part of India's Urban Malaria Scheme.^55^

Strategies for Integrated Vector Management (IVM)^56^ emphasize the need for the development of new insecticides, and the evaluation and implementation of alternative approaches because current options are limited. Where malaria vector populations are still susceptible to pyrethroids, that is, LA and the Pacific, it is essential to preserve future use through regular assessment of susceptibility status and, most importantly, the implementation of an appropriate IRM plan to minimize the risks of developing resistance.^57^

Few contemporary successful initiatives using alternative approaches as complementary control measures have been documented. Intensive environmental interventions to reduce *Anopheles* populations in semiarid environments in Eritrea^58^ and the initiatives in Mexico and Central America to stop the use of DDT,^59^ in which physical destruction of larval habitats or removal of filamentous algae associated with abundance of *Anopheles* larvae (i.e., *An. pseudopunctipennis*), led to an important reduction in the mosquito vector populations.^60^ In most countries, however, strategies against larval habitats are unlikely to be addressed due to the difficulty in identifying the breeding sites, site diversity, opportunistic use for most vector species of many water sources, and expense associated with and scale necessary for landscape modification.^61^ Furthermore, control methods against adult mosquito populations, particularly those which reduce their survival rate, may have a greater direct impact on malaria transmission. Unfortunately, few alternatives to existing insecticides are currently available^62^ and recommended for global incorporation in malaria control programs.

Resistance management strategies include rotation of insecticides with different modes of action and resistance mechanisms, or mosaic applications. However, it is becoming more common to find populations that have been exposed to different groups of insecticides from use in public health and/or agriculture, and the emergence of multiple resistance, or populations in which a resistance mechanism is causing cross-resistance to multiple compounds, especially if those compounds are in different classes of insecticides.

Usually, when results of the susceptibility bioassays indicate emerging or emergent resistance, malaria control program authorities make decisions regarding the necessary change of insecticides in that particular area. However, these bioassays do not provide any information on the strength of this resistance, and, because the correlation between results of diagnostic dose assays and control effectiveness remains undefined, simple detection of resistance in a mosquito population is often not sufficient evidence to implement a change in insecticide policy. Recently, an intensity test has been proposed to quantify the strength of resistance,^9^,^63^ which will give more information on the level of resistance that may lead to operational failure.

Besides physiological and genetic insecticide resistance, another challenge is behavioral resistance where vectors feed and rest outdoors as is seen with the main malaria vectors in LA and other regions.^64^,^65^ This behavior can be induced by extensive indoor vector control whereby previously endophilic species become more exophilic and avoid treated surfaces such as walls or bed nets. This is the case of *An. farauti* in Papua New Guinea^34^ where despite full physiological susceptibility to PY, a change in behavior to avoid surfaces with insecticides is creating an enormous challenge for malaria control. In Africa, outdoor transmission is a growing concern since major changes are taking place in which parasite transmission is shifting from the dominant and highly endophilic vector, *An. gambiae* s.s., to the more exophilic and outdoor-adapted vector, *An. arabiensis*.^66^ Also, new findings report entirely exophilic *An. gambiae* s.s. populations with high susceptibility to *P. falciparum*.^67^ Tools to effectively manage this outdoor and early evening transmission are urgently needed and although this behavioral trend may reduce insecticide resistance, it is a challenge for malaria control, particularly in countries moving toward malaria elimination.

As evident in the 10 ICEMR programs distributed throughout the malarious regions of the world, insecticide resistance is a growing and alarming problem for malaria control programs. Novel insecticides and alternative strategies are desperately needed for vector control, and would be better coupled with IVM and IRM programs. The hope is that future malaria control programs will have the tools to better integrate vector control with complementary antimalarials or vaccines that reduce or prevent parasite loads in hosts. Realistically, in the next 15 or more years, vector control will remain an essential component of malaria control programs.
